# Central venous catheter infections: building a causal model with expert domain knowledge to inform future clinical trials

**DOI:** 10.1186/s13756-025-01630-6

**Published:** 2025-10-08

**Authors:** Jessica A. Schults, Yue Wu, Thomas Snelling, Gladymar Pérez Chacón, Daner Ball, Karina Charles, Julie Marsh, Charlie McLeod, Hideto Yasuda, Claire M. Rickard

**Affiliations:** 1 Herston Infectious Diseases Institute, Metro North Health, Brisbane, QLD Australia; 2 School of Nursing, Midwifery and Social Work, The University of Queensland, St Lucia, QLD Australia; 3 Sydney School of Public Health, Faculty of Medicine and Health, The University of Sydney, Camperdown, NSW Australia; 4 Department of Infectious Diseases, The Children’s Hospital at Westmead, Sydney Children’s Hospitals Network, Westmead, NSW Australia; 5 Menzies School of Health Research, Charles Darwin University, Darwin, NT Australia; 6 Wesfarmers Centre of Vaccines and Infectious Diseases, The Kids Research Institute, Nedlands, WA Australia; 7 Centre for Child Health Research, University of Western Australia, Crawley, WA Australia; 8 Department of Emergency and Critical Care Medicine, Jichi Medical University Saimata Medical Center, Saitama, Japan; 9 Department of Clinical Research Education and Training Unit, Keio University Hospital Clinical and Translational Research Center, Tokyo, Japan; 10 Alliance for Vascular Access Teaching and Research, Griffith University, Brisbane, QLD Australia; 11 University of Queensland Centre for Clinical Research, Brisbane, QLD Australia

**Keywords:** Central venous catheter, CLABSI, Directed acyclic graph, Causal modelling, Infection control, Infection prevention, Clinical trial design

## Abstract

**Aim:**

Central venous catheters (CVCs) are essential for long-term therapies but carry a high risk of central line-associated bloodstream infections (CLABSIs), which significantly impact patient outcomes and healthcare costs. This study aimed to develop a causal model for CLABSI using expert knowledge to guide future clinical trials and prevention strategies.

**Methods:**

We constructed a directed acyclic graph (DAG) informed by literature and expert knowledge elicitation. A multidisciplinary team of clinicians, including infectious disease and vascular access experts, participated in interviews and workshops to refine the DAG, resulting in a final model with 30 variables representing CLABSI development.

**Findings:**

The expert-elicited DAG identified two main pathways, patient-related and CVC-related, each contributing to CLABSI risk. Variables and relationships in the DAG highlighted key patient characteristics, CVC management practices, and overlapping factors influencing infection. This model serves as a novel framework to understand CLABSI causation and supports trial design by identifying confounding factors, causal pathways, and meaningful endpoints.

**Conclusions/implications:**

Our causal DAG provides a structured representation of CLABSI risk factors, which may support the design of clinical trials examining interventions to reduce CVC-related infections. By clarifying causal mechanisms, the DAG can enhance the specificity of endpoints and improve the rigor of prevention strategies.

**Supplementary Information:**

The online version contains supplementary material available at 10.1186/s13756-025-01630-6.

## Introduction

Central venous catheters (CVCs) facilitate the administration of long-term therapies such as chemotherapy and parenteral nutrition, but their use increases the risk of bloodstream infections. Central line associated bloodstream infections (CLABSIs) place significant strain on global health services contributing increased risk of mortality, length of stay and healthcare costs [1–3]. CLABSI is associated with longer length of stays (difference 12.1–17.4 days dependent on intensive care unit requirement), higher costs ($25,207–$55,001 per admission) and a > 3.5 fold increased risk of mortality [4]. Risks are even higher in groups of either age extreme (old or young) or those with chronic disease and multiple morbidities [4]. International consumer surveys highlight CLABSI prevention as a top priority for health care consumers [5]. As CLABSIs become harder to treat [6] due to rising antimicrobial resistance [7, 8] their prevention has become a global public health concern.

Identifying factors which contribute to CLABSI events can serve to inform prevention initiatives. A directed acyclic graph (DAG) is a graphical representation of variables (e.g. prognostic factors, modifiers, intermediate outcomes), referred to as nodes, and their relationships with each other, indicated by directed arcs (arrows). A DAG is ‘causal’ when the arcs are taken to represent causal relationships between the connected variables [9, 10]. As a graphical representation of a complex health problem, DAGs can inform clinical trial design, including the selection of outcomes and endpoints for evaluation, and the identification of confounding variables [11]. Development of causal DAGs with subject domain experts can promote a more robust and realistic representation of the clinical problem based on existing evidence, expert knowledge and experience with the condition under study [11]. We therefore aimed to develop a causal qualitative expert DAG, using domain expert knowledge, to represent the causal processes underlying CLABSI development and to inform the design of future clinical trials and quality prevention activities. To our knowledge, this is the first such DAG for CLABSI.

## Methods for DAG development

A preliminary CLABSI DAG was constructed using a priori causal assumptions, established from published literature and the team’s extensive preliminary work [12–15]. CLABSI was defined in line with the US Centers for Disease Control and Prevention (CDC) a bloodstream infection (BSI) with an eligible organism (or repeated commensals), central venous catheter present on the day of (or the day before) the BSI event, and no other microbiological source [16]. We used iterative co-design methods to refine the initial DAG, using one on one interviews with domain experts (clinicians) [9, 17] followed by a knowledge elicitation workshop. A waiver of ethics was provided by the University of Queensland Human Research Ethics Committee.

Purposive sampling of clinical experts across countries (Australia, Japan, France), health disciplines (i.e., medicine or nursing) and clinical specialties (i.e. infectious diseases, anaesthesiology, haematology/oncology, and infection prevention) was undertaken via email advertisement through investigator networks (e.g., The Association for Vascular Access Research and Teaching Group, Herston Infectious Diseases Institute). Interviews utilised a semi structured interview guide and were conducted both online and in person, lasting approximately 45 min. On completion of the interviews we conducted a virtual knowledge elicitation workshop, presenting interview findings and the initial DAG to workshop participants. The aim of the workshop was to review pathways, influencing variables and endpoints. The workshop was facilitated by the Herston Infectious Diseases Institute Research Advisory Committee and included clinical and academic experts (subset of interview participants who expressed interest in further participation) in the fields of infectious disease, infection control and vascular access. Content analysis was used to analyse interview and workshop responses with the DAG and iteratively refine the associated variable dictionary, which required post workshops follow up emails. Limited further refinements and simplification of the expert-informed DAG were made by the authorship team, which includes causal methods experts (YW, TS, GPC). DAG results are reported in line with reporting guidelines [18].

## Results: final DAG description

**Fig. 1:**
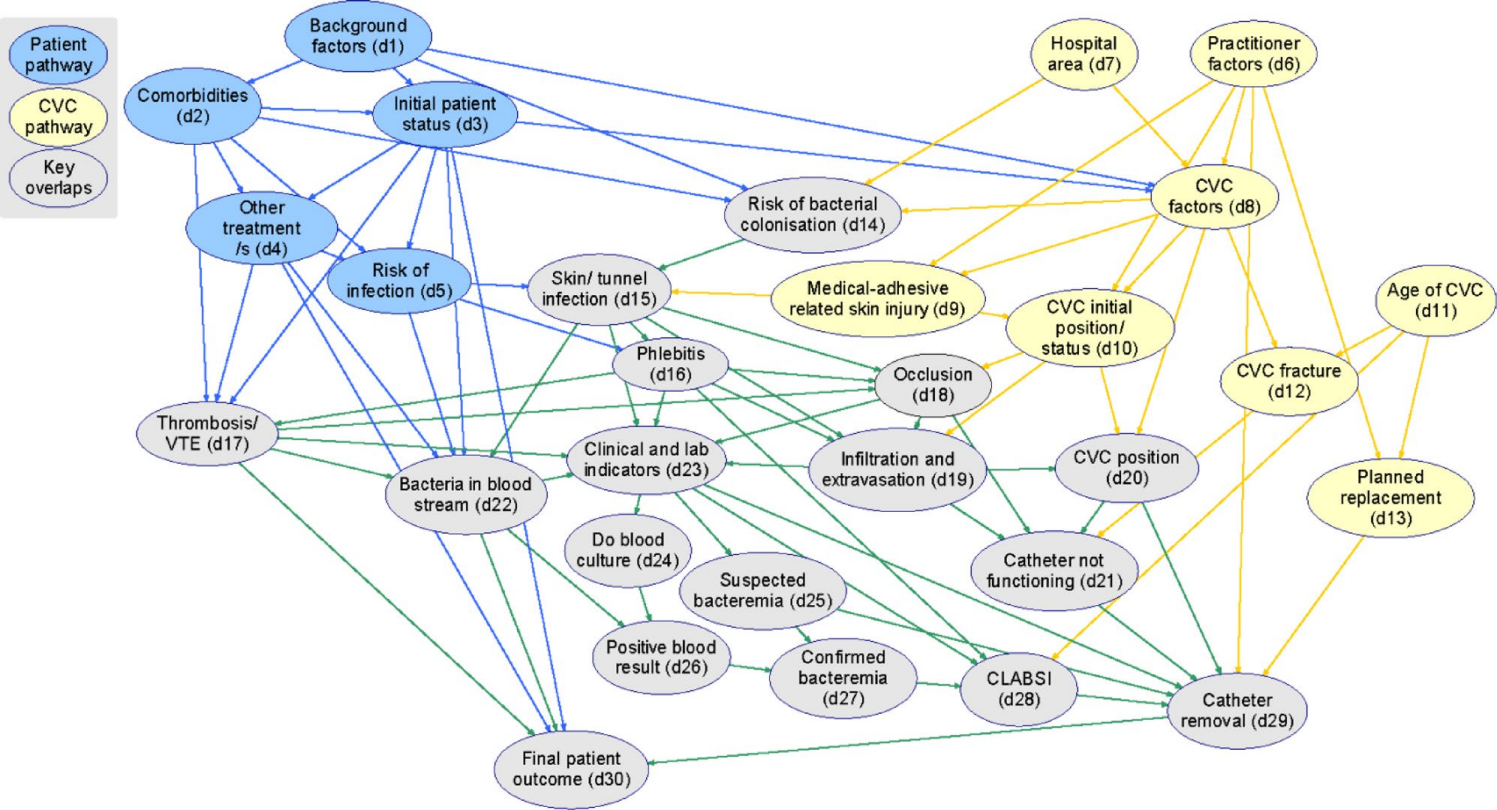
The expert-elicited DAG v1.2. Note: Numbers within the model nodes correspond with the narrative description. Yellow nodes: CVC variables; blue nodes: patient variables; grey nodes: overlapping variables. Arcs are coloured to indicate what pathways influence a node—patient pathway variables (blue), CVC pathway variables (yellow), overlapping pathways (green). The detailed model structure and definition for each variable are provided in Supplementary material 1

We conducted 11 interviews (3 nurses; 8 medical officers), with 17 participants at the knowledge elicitation workshop (6 nurses; 8 medical officers; 2 pharmacists; 1 genomic scientist). The final DAG is outlined in Fig. 1 alongside a variable dictionary, documenting the causal assumptions represented by each arc. The Expert DAG comprises 30 variables representing the mechanistic process of the development and diagnosis of CLABSI (Fig. 1). To capture the trajectory of CLABSI development, we outline key variables and predominant pathways over time, highlighted the patient pathway (in blue), CVC pathway (in yellow) and overlapping pathways (in grey and green). We start with two initial sets of variables, namely, the *patient-related pathway* (blue nodes d1–d3) and the *CVC-related pathway* (yellow nodes d6–d8, d11). For downstream (i.e. child) variables that are influenced by upstream (i.e. parent) variables in the same initial set, these remain in the previous defined pathway; otherwise, if the variable lies on both the patient and CVC-related pathway then they are considered an overlap variable. This colour-coded approach enables us to show how the initial set of patient- and CVC-related variables can causally drive CLABSI development in an interactive manner (grey). Supplementary material 1 provides a detailed DAG variable data dictionary.

A range of demographic factors (i.e., age, sex) and morbidities (i.e., immunocompromise) influence the risk of CLABSI [19]. We simplify the DAG by collapsing these factors into just two nodes: background factors (d1), and morbidities (d2). The patient pathway originates from the point of presentation to a healthcare facility. Influenced by various patient background factors (d1) and morbidities (d2), the initial patient status (d3) [20] gives rise to a dynamic disease and clinical management processes, including different CVC therapies (d4) and CVC-relevant (d8) decisions [19, 21]. These disease processes give rise to one or more signs, symptoms, and laboratory indicators (d23) of infection. The node final patient outcome (d30) reflects patient and disease progression for example complete recovery, persistent infection, death; that is how the initial patient status (d3) becomes improved or worsened depending upon the progressing disease processes (thrombosis/VTE (d17); bacteria in bloodstream (d22)), and the clinical management that occurred during the hospital admission, including CVC-related care.

The CVC-related pathway originates from a set of clinician-related (practitioner factors (d6), insertion environment (hospital area (d7)), and device-related factors (CVC factors (d8); medical adhesive related skin injury (d9); CVC initial position/status (d10)) [22]. However, the CVC-related pathway interacts with the patient-related pathway to mutually influence the risk of bacterial colonisation (d14) and skin/tunnel infection (d15), (i.e. most nodes are on the overlap pathway from this point onwards). The risk of CLABSI can be influenced by CVC-related factors (d8), practitioner-related factors (d6) and hospital area—insertion environment (d7) [23] via all pathways. Along each pathway there are mediating processes that influence CLABSI risk, such as the age of the CVC (i.e. dwell time; d11). In our DAG, catheter-related complications are represented as clinical and laboratory indicators (d23) [24]. The occurrence of these complications may directly influence the diagnosis of CLABSI (d28). These CLABSI-predisposing complications include development of a CVC-related thrombosis (d17 via the green arrow), CVC infiltration and extravasation (d19) [25], and medical adhesive skin related injury (d9) [26].

The risk of infection (risk of infection (d5)) is a key mediating mechanism between the patient’s initial status and downstream disease processes [22], because it can increase the probability of bacteria accessing and entering the blood stream (bacteria in bloodstream (d22))—the most important disease process within the context of CVC management. Infection, including bacteraemia, is a ‘latent’ event, meaning that it must be inferred indirectly (with varying confidence and potentially incorrectly) from the presence of various signs, symptoms or laboratory evidence (clinical and lab indicators (d23)), rather than being observed directly [27]. Such indicators are used to form a CLABSI diagnosis (CLABSI diagnosis (d28)) in practice. In addition to infection, thrombosis (bacteraemia/VTE (d17)) is an alternative disease mechanism that can predispose to CLABSI. Both infection and thrombosis can be influenced by patient and CVC-related factors [28–33], reflected by the number of green arrows entering their respective nodes (d17 and d22). The two disease mechanisms, as well as the therapy delivered through the CVC, can drive CVC complications. For example, thrombosis may increase the risk of occlusion (occlusion (d18)) [34, 35].

Due to the highly dynamic nature of this problem, attributing the CVC as the cause of the bacteraemia (bacteria in bloodstream (d22); i.e. CVC-dependent) is challenging. The operational definition of CLABSI diagnosis (CLABSI diagnosis (d28)) may lead to under ascertainment of CVC-dependent bacteraemia due to mild or non-specific clinical manifestations (clinical and lab indicators (d23) and suspected bacteraemia (d25)), failure to perform testing or poor test sensitivity (false negatives) (positive blood result (d26)) [27]. Conversely CLABSI incidence may be overestimated due to misattribution of infections arising independently of the CVC-related pathway via the patient pathway [36]. From the DAG perspective, the event of interest is presence of bacteria in the bloodstream (bacteria in bloodstream (d22)) mediated by pathways indicated by green arrows (skin/tunnel infection (d15), phlebitis (d16), thrombosis/VTE (d17) and originating from upstream nodes (e.g., medical adhesive related skin injury (d9)). CLABSI involves the presence of a pathogen, typically bacteria, in the bloodstream (bacteria in bloodstream (d22), originating from the patient’s environment, skin, or gastrointestinal tract [37]. A primary pathogenic route is via the catheter insertion site due to compromised skin integrity [24, 38] (d9) potentially exacerbated by poor inserter technique [23], inadequate pre-insertion skin decontamination, re-colonisation of skin flora post-insertion, or non-sterile/non-intact dressings or securement devices [39–41]. Another pathogenic route is via the internal lumen of the CVC and originating from clinical staff’s hands or the environment [42]. Diagnosis of CLABSI (d28) is informed by clinical assessment and indicators (d23), and/or patient risk factors (d1) [27, 43]. The ‘clinical and laboratory’ node acts as a key collider where multiple patient-related and CVC-related causal pathways converge. As more evidence accumulates, a provisional diagnosis of CLABSI may be made, prompting laboratory blood culture testing (blood culture (d24)) and/or catheter removal due to suspected bacteraemia (d25) without further investigation. Interpretation of the blood culture results (positive blood culture (d26)), including the organism type and colony density, may confirm bacteraemia (d27) and exclude contamination. The isolation of a compatible organism, without evidence of a concurrent non-CVC source, is suggestive of a causative relationship with the CVC. This finding is reported as significant in the laboratory report (clinical and lab indicators (d23)), with antimicrobial susceptibility results [21]. A diagnosis is made when the blood culture result is received, directly influencing subsequent decisions regarding catheter removal (d29) and the final patient outcome (d30).

## Discussion

The challenge of preventing, diagnosing and managing CLABSIs prevention, diagnosis and management is complicated by ambiguity in case definition and pathogenic mechanisms. This work represents the first step in developing an explicit explanatory model to depicts the causal pathways involved in CLABSI development. In our model, CLABSI arises from two broad causal pathways representing patient-related and CVC-related factors. In line with current recommendations for CLABSI prevention, future trial interventions may therefore be broadly divided into those that target one of these pathways. Interventions including ultrasound guided insertion, sutureless securement, and removal of unnecessary catheters [24, 44–47].

In other fields, DAGs have been used to inform (1) analysis plans, (2) trial end points [48], and (3) decision support algorithms [9]. One example of this is a recent qualitative expert DAG, developed to define the mechanistic actions for urinary tract infection (UTI) in children. The resulting DAG was then used to inform the development of an applied Bayesian network to support the diagnosis of UTI. A motivation for the current DAG, is to inform the design of CLABSIs prevention trials. The DAG helps to evaluate the strengths and limitations of alternate trial endpoints impacted by the studied intervention [49]. For example, confirmed bacteraemia (d27) in patients with CVCs may not be causally attributable to the CVC, and therefore might not represent true CVC-dependent harm. In a trial of prevention strategies, an endpoint definition of CLABSI which is non-specific for CVC-dependent bacteraemia could increase the risk of failing to detect a clinically important effect; endpoint specificity might be improved by including additional evidence supporting a CVC-related source. The DAG also indicates that apart from bacteraemia, any CVC-related intervention could plausibly affect the risk of other important harms (e.g. thrombosis (d17) and phlebitis (d16)) and therefore it may be important to also capture these endpoints.. The need for CVC removal (d29) can arise as a downstream consequence of a range of CVC-dependent harms occurring either alone or in combination (e.g. thrombosis, phlebitis or bacteraemia). Because CVC removal represents a single downstream event, it may have appeal as a single ‘summary’ endpoint for a range of possible harms when comparing CVC-related interventions. However, insofar as the need for CVC removal may be subjective, influenced by practitioner factors (d6). Additional factors such as ‘planned’ replacement (d13) also makes it a non-specific endpoint. If CVC removal is to be used in future trials as an endpoint, it may be necessary to improve its specificity, this can be achieved by delineating the endpoint as unplanned removal, necessitated by a complication. B. The DAG also helps identify factors (e.g. presence of morbidities (d2) and/or the patient’s status on presentation (d3)) which may be predictors of the endpoint which must be balanced across trial groups (e.g. by stratified randomisation) with appropriate adjustment in the statistical analysis. Further the DAG can be used to select end-user important outcomes for infection prevention trials of catheter interventions (e.g., intraluminal or extraluminal) to prevent CLABSI or inform the clinical decision tools, e.g., early prediction of CLABSI in the clinical environment.

Macro-level interactions (system level factors), such as the availability of infection prevention and control personnel, may modulate CLABSI risk, however we did not include these as explicit nodes for two reasons. First, the primary goal was to depict the *mechanistic* interaction between patient-level factors and CVC care processes to support the selection and definition of trial endpoints. Inclusion of all upstream determinants would have made the DAG visually dense without improving its value for the primary purpose of designing a clinical trial with randomisation at the level of the individual, rather than the cluster (i.e. hospital). Instead, such determinants were grouped into existing higher-order nodes, for example D1/D2 (background factors and comorbidities), D3 (initial clinical status), and D6 (practitioner factors). Second, causal DAG methodology allows *pragmatic extension*: if future analyses target policy-level questions (e.g., the impact of nurse-to-patient ratios on CLABSI), these macro-level variables can be layered onto the current structure without altering the core patient-device mechanisms depicted here. Excluding macro-level factors, such as staffing or institutional resources, may lead to residual confounding at the cluster level. Therefore, future work will address this by expanding the current DAG to capture potential mechanistic impacts from macro-level factors that are relevant to CLABSI occurrence under the research specific questions (e.g., use of vascular access teams) [50].

The developed DAG is a simplification of real-world complexity, and we acknowledge it is likely incomplete. Concepts such as CVC-related factors (d8) and infection risk (d5) are intentionally broad and will likely require further detailed exploration in subsequent, study-specific contexts. By explicitly stating assumptions, we enable testing and targeted discussion among end-users. Further, we recognise that broader macro-level influences, such as staffing and institutional factors, might not be adequately captured (for future trial/practice needs) within the DAG, potentially influencing the outcomes observed. Finally, the DAGs inherent complexity may lead to challenges in clinical application, with the aggregation of variables into higher-order nodes potentially minimising important clinical covariates. While the DAG does not propose solutions, it forms a basis for rational consideration of these trial design elements. By quantifying the strength of the arcs in the causal DAG (as a Bayesian network) using either empiric data or expert-elicited probabilities, trialists might better understand the consequences of these design choices.

## Supplementary Information

Below is the link to the electronic supplementary material.

Supplementary Material 1

## Data Availability

No datasets were generated or analysed during the current study.

## Abbreviations

CVC: Central venous catheter
CLABSI: Central line-associated bloodstream infection
DAG: Directed acyclic graph
CDC: Centers for Disease Control and Prevention
BSI: Bloodstream infection
ICU: Intensive care unit
US: United States
