# Supplementary material for: Central venous catheter infections: building a causal model with expert domain knowledge to inform future clinical trials
Source: Antimicrob Resist Infect Control. 2025 Oct 8;14:116. doi: 10.1186/s13756-025-01630-6 (PMC12506371; doi:10.1186/s13756-025-01630-6)
Supplement: Supplementary file 1 — Supplementary Material 1 [file 13756_2025_1630_MOESM1_ESM.docx]

## Supplementary material 1. DAG dictionary

| **ID** | **Variable Item** | **Description** | **Parent nodes within the putative DAG** | **Assumed causal relationship with parent nodes** | **Observability status** |
| --- | --- | --- | --- | --- | --- |
| d1 | Background factors | Patient, societal and environment factors that may contribute to the clinical problem of interest, *i.e.*, central venous catheter (CVC) and intravenous catheter infections. | None | Not applicable | Observable, however, often not exhaustively observed |
| d2 | Morbidities | The presence of diseases or medical conditions that occur prior to presentation to the hospital, such as chronic thrombophilia and immunocompromise. | Background factors (d1) | Patients with distinct background disease, social and environment factors have different risks at developing morbidities. However, explicit mechanisms behind such variation are complex and beyond the scope of this DAG. | Observable |
| d3 | Initial patient status | Patient’s initial disease status at hospital presentation such as cancer and sepsis and their associated clinical manifestations, which can be recorded as reason for presenting to hospital. | Background factors (d1), Morbidities (d2). | Distinct background factors can drive acquisition of different diseases. Morbidities could increase the complexity of the patient’s disease status. | While the underlying disease process is latent (not observable), clinical indicators and diagnoses are usually observed (recorded). |
| d4 | CVC therapy | Drugs or fluids delivered via the CVC, e.g., fluids, chemotherapy, | Morbidities (d2), Initial patient status (d3). | Patient with morbidities and more complex initial status requires extra and a multidisciplinary approach to care. This can drive clinician’s decisions about treatment. | Observable |
| d5 | Risk of infection | This is a summary concept that describes the patient’s total risk of infection at the time of hospital presentation. | Morbidities (d2), Initial patient status (d3), CVC therapy (d4) | Morbidities such as immunocompromise and certain initial disease status can make the patient more susceptible at developing infection. If managed with appropriate therapy, the risk of infection can be reduced. | Latent |
| d6 | Practitioner factors | Characteristics or experience of the clinician who’s responsible for selecting and inserting the CVC device. | None | Not applicable | Observable |
| d7 | Hospital area | Hospital locations where CVCs may be inserted, e.g., operating theatre, radiology, emergency, intensive care unit. | None | Not applicable | Observable |
| d8 | CVC factors | All CVC device specific factors including, material, size, location of insertion, number of lumens, securement and decontamination measures. We summarise this as a broad concept for the purpose of this DAG, and it needs to be stepped out to facilitate investigation of specific intervention that targets different aspects of the CVC device. | Background factors (d1), Initial patient status (d3), Hospital area (d7), Practitioner factors (d6). | Depending on the specific characteristics, different CVC devices may be available at different hospital areas. The practitioner may have a preference of certain CVC device due to their past experience, or based on their assessment of a patient’s background factors and initial status. | Observable |
| d9 | Medical-adhesive related skin injury | An injury to the skin from a medical adhesive (e.g. CVC adhesive dressing). | CVC factors (d8), Practitioner factors (d6). | Practitioner’s experience and CVC factors such as size and securement may influence how the device gets inserted thus the chance of skin injury. | Observable |
| d10 | CVC initial position/ status | The initial position and status of the CVC when placed. This is usually confirmed on chest x-ray. | CVC factors (d8), Practitioner factors (d6), Medical-adhesive related skin injury (d9). | Practitioner’s experience and CVC factors such as size and securement may influence how the device gets inserted thus the initial position of the CVC. The presence of medical-adhesive related skin injury can make the initial position less stable. | The true underlying position is latent. However, that status may be inferred through imaging technology. |
| d11 | Age of CVC | How long the CVC has been in place, or the duration of use. | None | Not applicable | Observable |
| d12 | CVC fracture | Breakage to or rupture of a CVC. | CVC factors (d8), Age of CVC (d11). | Certain CVC material may be more susceptible to fracture. The chance of facture will increase as the device gets used for longer (i.e., age of CVC). | Observable |
| d13 | Planned replacement | Routine maintenance and replacement of the CVC device in use, usually informed by ward’s procedural guideline. | Age of CVC (d11), Practitioner factors (d6) | Ward’s procedural guideline may specify how long a CVC device should be used, which may be change (made shorter or longer) upon Practitioner factors/ preference. | Observable |
| d14 | Risk of bacterial colonisation | This is a summary concept that describes the total risk of bacterial colonisation around CVC insertion site at the time of initial insertion (extraluminal infection risk). | Background factors (d1), Morbidities (d2), Hospital area (d7), CVC factors (d8) | Background factors and morbidities may lead to various degrees of exposure to bacteria, as well as a patient’s susceptibility to colonisation. The environmental microbiome can vary in different hospital areas. And bacteria may more or less likely to colonise depending on distinct CVC characteristics (e.g., material). | Latent |
| d15 | Skin/ tunnel infection | An infection of the catheter entry point (skin) or catheter tunnel/pathway. | Risk of infection (d5), Risk of bacterial colonisation (d14), Medical-adhesive related skin injury (d9). | Presence and high volume of bacterial colonisation can serve as the source of skin/ tunnel infection, and an infection is more likely to establish if skin injury is present. Patient’s risk of infection can also contribute to the skin/tunnel infection, either via a higher susceptibility to developing infection, or due to existing underlying infection (e.g., bacteria circulating in the bloodstream) | Latent |
| d16 | Phlebitis | Inflammation of a vein near the skin surface, which can be indicated by certain clinical symptoms, signs and laboratory markers. | Risk of infection (d5), Skin/tunnel infection (d15) | The inflammation of the vein may establish due to an expansion of the skin/ tunnel infection or other existing infections. It may also worsen when a patient is at a high risk (e.g., susceptibility) to infection. | Latent |
| d17 | Thrombosis/ VTE | Thrombosis or venothromboembolism, a blood clot that limits/blocks blood flow within the veins. | Initial patient status (d3), Morbidities (d2), CVC therapy (d4), Phlebitis (d16). | Certain morbidities and initial patient status may lead to a higher chance of developing thrombosis. Different therapies may have various impact on thrombosis, some preventive and some increases it due to side effects. Established phlebitis can cause formation of blood clot. | Latent, although its diagnosis can be observable. |
| d18 | Occlusion | The blockage of a blood vessel. | Thrombosis/VTE (d17), Skin/tunnel infection (d15), Phlebitis (d16), CVC initial position/status (d10), CVC therapy (d4). | Blood vessel can get blocked due to various reasons, including clots (d17), inflammation (d15 and d16), poor CVC position (d10), and medical solutions delivered through the device (d4). | Latent |
| d19 | Infiltration and extravasation | The leakage of a medical solution from a vein into its surrounding tissue. | Skin/ tunnel infection (d15), Phlebitis (d16), Occlusion (d18), CVC initial position/ status (d10). | The leakage would occur when there is a breakage of the vein, which can be caused by inflammation (d15, d16), blockage (d18) and malposition of the tip of the device (d10). | Latent |
| d20 | CVC position | The position of the CVC at a later time point after the initial insertion. Abnormal position includes tip malposition, or more extremely, dislodgement. Usually confirmed on chest x-ray. | CVC factors (d8), CVC initial position/status (d10), Infiltration and extravasation (d19) | Tip malposition or dislodgement at a later time point more likely to occur if the quality of original/initial position was poor (d10), potentially for certain CVC characteristics (d8, e.g., securement), and if [needing help for d19] | Latent. However, it may get clearly inferred as the degree of malposition becomes high or extreme. |
| d21 | CVC not functioning | CVC stops functioning as intended (for delivering medical solution), and this can be defined as CVC failure. | Occlusion (d18), Infiltration and extravasation (d19), CVC fracture (d12), CVC position (d20) | The intended delivery of medical solution can be interrupted when there is delivery to the wrong place (d20), solution cannot come through (d18) or solution leaks (d12 and d19). | Observable |
| d22 | Bacteria in bloodstream | The presence of bacteria in the bloodstream. Note this refers to the true status of the world that is not directly observable. | Initial patient status (d3), Risk of infection (d5), CVC therapy (d4), Thrombosis/VTE (d17), Skin/tunnel infection (d15), Phlebitis (d16). | For a given patient, bacteria are more likely to be present in their blood stream if the patient has pre-existing/underlying infection (d3), a high baseline risk of infection(d5), has developed bacterial skin/tunnel or vein infection (d15-16), or has developed a thrombus affecting the CVC producing a nidus for bacterial attachment (d22). If managed appropriately (d4, e.g., with antibiotics), the probability of bacteria in bloodstream may be reduced. | Latent |
| d23 | Clinical and lab indicators | Clinical and lab indicators are signs, symptoms or measures used to diagnose suspected bacterial infections which may or may not be relevant to CVCs. | Thrombosis /VTE (d17), Infiltration and extravasation (d19), Skin/tunnel infection (d15), phlebitis (d16), Occlusion (d18), Bacteria in blood stream (d22), | Any underlying disease processes (d15-d19, d22) can give rise to clinical and lab indicators, including those are non-specific (e.g., fever) and more specific (e.g. erythema around the insertion site). | Observable |
| d24 | Do blood culture | Blood culture taken from the patient for suspected blood steam infection. | Clinical and lab indicators (d23) | A decision to perform blood culture can be made based on observations of clinical and lab indicators. | Observable |
| d25 | Suspected bacteraemia | The clinician suspects that bacteria may be present in a patient’s bloodstream, but this is yet to be confirmed by blood culture. | Clinical and lab indicators(d23) | Clinician’s suspicion of bacteraemia is based on compatible clinical and lab indicators. | Observable |
| d26 | Positive blood result | Positive blood culture result which indicates the presence of bacteria in the blood, however, it could also be due to contamination. | Do blood culture (d24), Bacteria in blood stream (d22) | Positive culture result requires a blood culture test is performed, and positive result is more like when bacteria is present in the blood stream (thus the culture sample). | Observable |
| d27 | Confirmed bacteraemia | Bacteraemia has been confirmed by a positive blood culture, plus additional clinical indicators for suspected bacteraemia. | Positive blood result (d26), Suspected bacteraemia (d25) | Confirmed bacteraemia is operationally define by a clinically suspected bacteraemia and positive blood result. | Observable |
| d28 | CLABSI diagnosis | Diagnosis of Central Line-Associated Bloodstream Infection. Deterministic definition of CLABSI specific to study and commonly used in the research field. It is intended to be highly specific for bacteremia which is attributable to the presence of a CVC, although neither sensitivity nor specificity will be perfect. | Age of CVC (d11), Clinical and lab indicators (d23),  Confirmed bacteraemia (d27) | Indicated by certain clinical symptoms, signs and laboratory markers (d23), CLABSI diagnosis is operationally defined as a laboratory confirmed bloodstream infection (d27, this excludes mucosal barrier injury bloodstream infection) with a vascular catheter in place >48h/2 calendar days (d11), and no other infection source (based on assessment of clinical indicators). | Observable |
| d29 | Catheter removal | The removal of a CVC. | Practitioner factors (d6), Planned replacement (d13), CVC position (d20), Catheter not functioning (d21), Clinical and lab indicators (d23), Suspected bacteraemia (d25), CLABSI diagnosis (d28) | A CVC device can be removed due to practitioner’s preference (d6), procedural guideline for routine maintenance (d13), functional concern (d20-21), suspicion or confirmation of infection or CLABSI (d23, d25, d28) | Observable |
| d30 | Final patient outcome | The final patient disease/health status, e.g., recovery or death. | Initial patient status (d3), Bacteria in blood stream (d22), CVC therapy (d4), Thrombosis (d17), Catheter removal (d29), | Initial disease status/severity (d3) and their progression since presentation (d22, d17) can influence on how a patient recover. This process can be further intervened by treatment received, or if the treatment gets interrupted due to catheter failure. | Observable |
